# Follow-up of patients with Hodgkin's disease following curative treatment: the routine CT scan is of little value

**DOI:** 10.1038/sj.bjc.6601052

**Published:** 2003-07-29

**Authors:** E T Dryver, H Jernström, K Tompkins, R Buckstein, K R Imrie

**Affiliations:** 1 The University of Toronto, Toronto, Ontario, Canada M5S 1A1; 2 The Jubileum Institute, Department of Oncology, Lund University Hospital, S-22185 Lund, Sweden; 3 Toronto Sunnybrook Regional Cancer Centre, 2075 Bayview Avenue, Toronto, Ontario, Canada M4N 3M5; 4 Sunnybrook & Women's College Health Sciences Centre, 2075 Bayview Avenue, Toronto, Ontario, Canada M4N 3M5

**Keywords:** Hodgkin's disease, follow-up, relapse, computed tomography scan

## Abstract

A total of 10–40% of patients with Hodgkin's disease relapse following initial curative therapy. Intensive follow-up is resource intensive and may identify false relapses. We performed a retrospective review of all patients with Hodgkin's disease treated at our centre between 1990 and 1999 to evaluate the utility of the components of follow-up. A total of 107 patients met the inclusion and exclusion criteria. The median age was 33 years and the median duration of follow-up 38 months. The total number of follow-up visits was 1209 and total number of CT scans 283. There were 109 suspected relapses of which 22 proved to be true relapses. Of the latter, 14 were identified clinically, six radiologically and two via lab testing. The routine CT scan detected only two relapses (9%), yet accounted for 29% of the total follow-up costs. Based on data from our centre, the cost per true relapse was $6000 US, 49% incurred by radiological tests. The majority of the cost of follow-up was incurred by routine follow-up (84%) as opposed to the investigation of suspected relapses (16%). We conclude that most true relapses are clinically symptomatic and that the routine CT is an expensive and inefficient mode of routine follow-up.

The majority of patients with Hodgkin's disease are now cured with primary therapy (Urba and Longo, 1992). Nevertheless, 10–15% of patients with localised disease and up to 50% of patients with advanced stage disease still relapse following initial curative therapy (Hasenclever and Diehl, 1998). Patients who relapse after radiation alone can be effectively salvaged with chemotherapy and a proportion of patients who fail after chemotherapy will be long-term survivors after stem cell transplantation (Linch *et al*, 1993).

Given the availability of potentially curative second-line therapy and the theoretical benefit of early detection and treatment of Hodgkin's relapses, most centres have adopted a policy of close follow-up following curative therapy for Hodgkin's disease. However, close follow-up is expensive as well as resource intensive and it may identify numerous suspected but false relapses, leading to further testing and heightened patient anxiety.

The follow-up protocols for most clinical trials are based on a report generated in 1989 by pathologists, radiologists and oncologists who had gathered in the Cotswolds (Lister *et al*, 1989). This report recommended that patients be assessed every 3 months during the first 2 years post-therapy, every 4 months during the third year, every 6 months during the fourth and fifth years, and annually thereafter. The authors recommended that the frequency and type of radiological studies reflect the initial sites of disease, but more specific guidelines were not provided. The uncertainty about the value of routine radiological investigations in the detection of relapse has increased after two studies showed that most relapses are detected as a result of patient-reported symptoms, not radiological investigations (Radford *et al*, 1997; Torrey *et al*, 1997).

We performed a retrospective review of all adult patients followed at the Toronto Sunnybrook Regional Cancer Centre between 1990 and 1999 post curative therapy for Hodgkin's disease. The objective of our study was to evaluate the utilities of the clinical assessments, the radiological tests and the laboratory tests to detect Hodgkin's relapses.

## METHODS

### Inclusion and exclusion criteria

We identified all adult patients with classical Hodgkin's disease (*n*=187) followed at our centre (which services a large metropolitan area with a population of 2.5 million people) between 1990 and 1999 using the centre's database. We excluded patients who were diagnosed with Hodgkin's before January 1st 1990 (*n*=10), who were not treated with curative-intent (*n*=13), who failed to respond to initial therapy (*n*=3) and those who were not followed exclusively at our centre for at least 12 months unless this follow-up was interrupted by death or relapse (*n*=54). Follow-up data were gathered on the remaining 107. Data were censured when follow-up ceased to be exclusively at our centre.

### Follow-up protocol

Patients were assessed every 3 months for the first 2 years after completion of therapy, every 6 months for years three to five inclusive, and annually thereafter. Assessments included clinical assessment (history and physical), a chest X-ray and a complete blood count. Other radiological testing including computed tomography (CT) scanning and gallium scanning were left to the discretion of the investigator.

### Outcomes

We defined a suspected relapse as a situation in which a clinical, radiological or laboratory abnormality prompted an intensification of follow-up, for example, an additional follow-up visit or an additional radiological investigation. A true relapse was defined as a situation in which a biopsy confirmed Hodgkin's relapse or in which a radiological finding was sufficient to warrant initiation of second-line therapy for Hodgkin's. A false relapse was defined as a suspected relapse followed by resumption of routine follow-up.

Suspected relapses were attributed to the patient when the patient expressed concern about a symptom or physical finding that prompted the physician to depart from the usual follow-up protocol. The suspected relapse was attributed to the physician's assessment when the physician arranged for intensification of follow-up based on the history or physical exam in the absence of patient concerns. Suspected relapses were attributed to radiological or laboratory testing when abnormal radiological or laboratory findings, respectively, led to a departure from the follow-up protocol. In a number of cases, more than one follow-up modality raised the possibility of relapse. In these cases, the suspected relapse was attributed to the least invasive modality beginning with the patient, physician, radiological and finally the laboratory testing. This hierarchical grading system has been used in a previous analysis of Hodgkin's follow-up (Torrey *et al*, 1997).

The following information was recorded for each patient: number of follow-up visits, chest X-rays, CT scans, total number of radiological investigations, number of suspected relapses and whether a true relapse occurred. For each suspected relapse, the modality that triggered the intensification of follow-up was identified and the number of additional follow-up visits and radiological investigations over and beyond the routine follow-up protocol were recorded. We calculated the international prognostic score (IPS) (Hasenclever *et al*, 1998) for all patients based on the blood work preceding the onset of therapy most closely. When blood work was missing (most commonly the pre-therapy albumin), we calculated the score as though the missing variables were normal.

The first follow-up visit was defined as the first visit after completion of therapy. Visits and tests unrelated to potential Hodgkin's recurrence were not included. We recorded all radiological investigations carried out after completion of therapy to detect relapse. Tests were recorded as routine when there was no indication in the physician's notes that a relapse was suspected. On the other hand, a test was attributed to a suspected relapse (and the modality that prompted this suspected relapse) when the physician's notes indicated that it would not otherwise have been performed. Once a relapse was suspected, all subsequent radiological investigations were attributed to that relapse until the resumption of the usual follow-up regimen. A CT of the chest, abdomen and pelvis was counted as one CT when performed on the same day. The ordering of CT scans was not directly limited by patient or provider financial considerations.

Follow-up information was collected until a biopsy showed relapse or a radiological finding prompted the initiation of second-line therapy, the patient died, the patient ceased to follow-up exclusively at our centre or until the last documented visit and radiological test.

### Cost calculations

The centre cost of clinic visits was derived from the centre's annual budget. Physician costs were determined using the provincial physician fee schedule. Costs of radiological testing were calculated by adding the professional and radiologist interpretation fees. The costs of a clinic visit including oncologist fees and the cost of a complete blood count were US $54, the cost of a chest radiograph was US $21 and the cost of a CT scan was US $174.

### Statistical analysis

Results were analysed using the Statistical Package for the Social Sciences (SPSS) version 10. The *χ*^2^ were used to perform simple tests of ratio unheterogeneity. Binary logistic regression models were used to analyse the associations between non-binary factors and the presence of a true relapse or one or more false relapses. Linear regression models were used to study the associations between the number of false relapses and other factors. All *P*-values were two-sided. Correlations between factors were analysed using the Pearson method.

## RESULTS

### Patient characteristics

Baseline characteristics are summarised in Table 1

**Table 1:** Characteristics of the 107 patients that met inclusion and exclusion criteria

*Demographics*		
Median age (range)	33	(17–82)
Male gender	61	(57%)
		
*Ann arbor stage*		
I	31	(29%)
II	60	(56%)
III	7	(6%)
IV	9	(8%)
B symptoms	27	(25%)
		
*Grade*		
Nodular sclerosis	66	(62%)
Lymphocyte depletion	1	(1%)
Mixed cellularity	20	(19%)
Lymphocyte rich	20	(19%)
		
*Treatment*		
Radiotherapy alone	55	(51%)
Combination therapy	34	(32%)
Chemotherapy alone	18	(17%)
		
*International prognostic score*		
0	23	(21%)
1	49	(46%)
2	23	(21%)
3	8	(8%)
4	2	(2%)
5	2	(2%)

. A total of 107 patients were included in our analysis, 61 men and 46 women. The median age was 33 years (range 17–82 years). In all, 54 patients were excluded because their follow-up was not carried out exclusively at our centre. This patient group did not differ significantly from the study population with regard to age and gender. A total of 51% percent of patients were treated with radiation alone, 17% with chemotherapy alone and 32% with combined modality therapy. The chemotherapeutic regimen was ABVD in 89% of patients. The remainder received MOPP or MOPP-ABVD.

### Follow-up logistics

Follow-up data are summarised is Table 2

**Table 2:** Follow-up logistics

	**Total**	**Median**	**Range**
Follow-up duration	4655 months	38 months	1–120 moths
No. of follow-up visits	1209	11	0–31
No. of CXRs	583	5	0–15
No. of CTs	283	2	0–10
No. of radiological investigations	977	9	0–27

This table illustrates the total and median number of visits and radiological investigations carried out during the follow-up of the 107 patients as well as median and total duration of follow-up.

. The total duration of follow-up was 4655 patient-months (388 patient-years). The median duration of follow-up was 38 months (range 1–120 months). The total number of follow-up visits was 1209 (median 11/patient) and the total number of radiological tests was 977 (median 9/patient). Eight haematologists/medical oncologists and four radiation oncologists were involved in the follow-up process.

### Suspected relapses and true relapses

Figure 1

**Figure 1:**
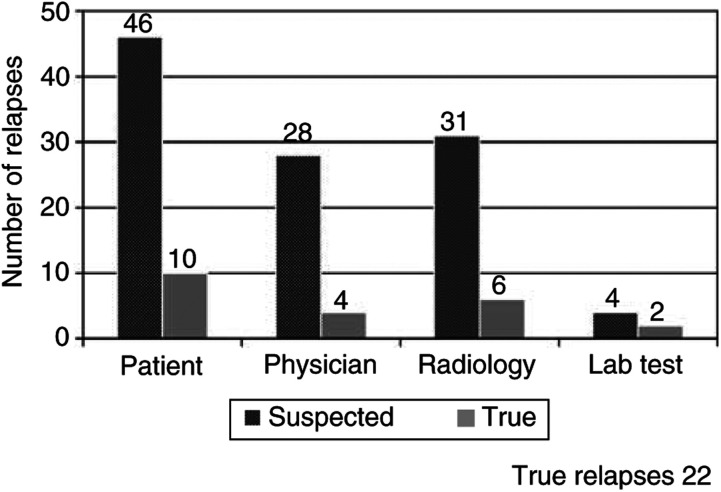
Suspected relapses and true relapses according to modality of detection. In 109 cases, an abnormality prompted an intensification of follow-up (suspected relapse) which led to the detection of Hodgkin's relapse (true relapse) in 22 cases. Suspected relapses were attributed to the ‘patient’ (46 cases, yielding 10 relapses) when triggered by a patient-reported symptom or sign; to the ‘physician’ (28 cases, yielding four relapses) when triggered by the physical examination; to ‘radiological’ (31 cases, yielding six relapses) when triggered by an abnormality on a CXR, CT or other radiological investigation; or to ‘laboratory’ (four cases, yielding two relapses) when brought on by an abnormal CBC. A hierarchical classification system was employed when multiple follow-up modalities suggested a possible relapse (see Materials and Methods).

illustrates the breakdown of the suspected and true relapses according to detection modality and Figure 2

**Figure 2:**
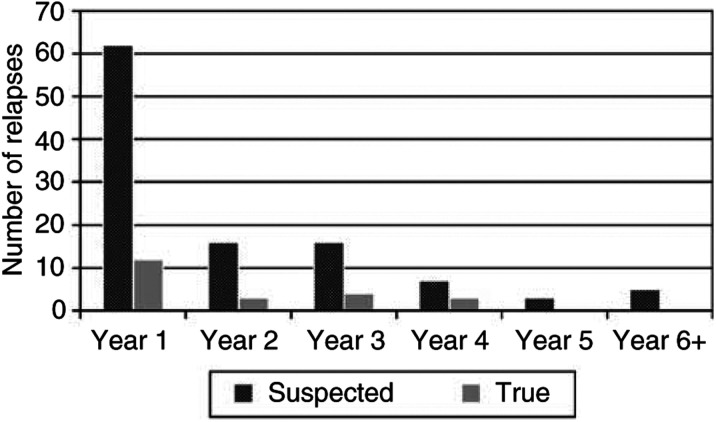
Suspected relapses and true relapses according to year of follow-up. This graph illustrates the breakdown by year of follow-up of the 109 suspected relapses, of which 22 were true relapses.

shows the breakdown by year. Relapse was suspected in 109 instances involving 68 patients. Relapse was suspected as a result of patient concerns in 42%, physician concerns in 26%, radiological findings in 28% and laboratory abnormalities in 4%. True relapses occurred in 22 patients. Of these, the largest proportion stemmed from patient concerns (10), four from physician concerns, six from radiological abnormalities and two from laboratory abnormalities. Of the six relapses identified radiologically, four stemmed from a routine chest X-ray and two from a routine CT. Of the 25 false relapses identified radiologically, seven stemmed from a routine chest X-ray, 12 from a routine CT, five from a routine gallium scan and one from an ultrasound. The outcomes of routine clinical, radiological and laboratory investigations are provided in Table 3

**Table 3:** Outcomes of routine clinical, radiological and laboratory follow-up

	**Clinical**	**CXR**	**CT**	**CBC**
Routine investigations	1038	544	211	1038
True relapses	14	4	2	2
False relapses	60	7	12	2

This table provides the total number of routine clinical follow-up visits, chest X-rays (CXR), computed tomography (CT) scans and complete blood counts (CBC). It also provides the number of true relapses identified by these follow-up modalities as well as the number of false relapse investigations that they generated. Patient- and physician-derived relapses were considered jointly. Six of the 109 suspected relapses were generated by radiological investigations other than CXR or CT (e.g., gallium scan).

.

### Factors predicting relapse

Ann Arbor stage IV (OR 8.44; 95% CI 1.57–45.4; *P*=0.013) and therapy other than combined modality treatment (OR 5.88; 95% CI 1.28–25; *P*=0.02) were associated with an increased risk of relapse. Among patients treated with chemotherapy, a higher IPS was associated with an increased risk of relapse (OR 2.10; 95% CI 1.08–4.07; *P*=0.03). The presence of prior suspected relapses did not affect the likelihood of a true relapse.

### Factors predicting the outcome of a suspected relapse

We analysed the settings of all first suspected relapses to determine which factors predicted the outcome of a suspected relapse. A suspected relapse was more likely to be a true relapse in patients treated with radiotherapy alone (OR 5.76; 95% CI 1.14–29.1; *P*=0.03). Age, gender, the presence of B symptoms, Ann Arbor stage, prognostic score and the time elapsed between the end of therapy and the suspected relapse did not help distinguish a true from a false relapse. The modality of suspected relapse was also unhelpful.

### Costs

**Figure 3:**
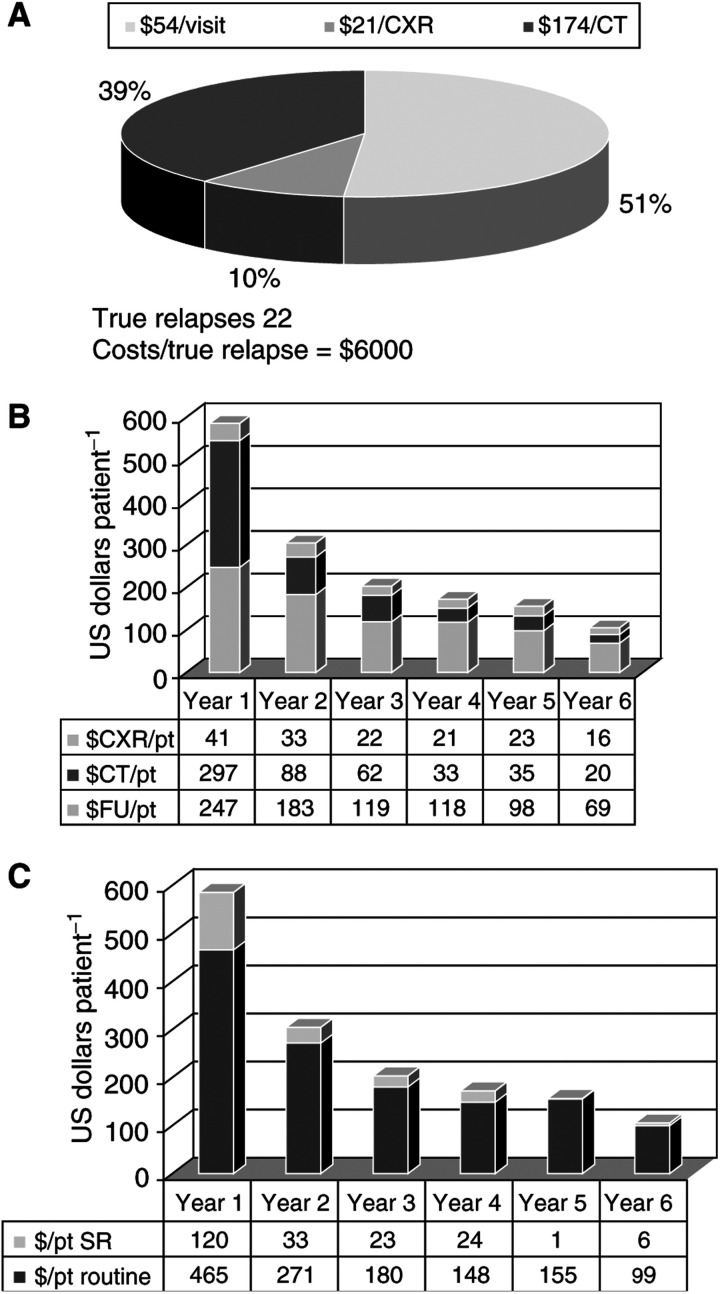
(**A**) Costs of follow-up. This figure illustrates the breakdown of the costs of follow-up. A total of 51% of the costs were attributable to the follow-up visits (including the routine CBC), 39% to the CT scans and 10% to the CXRs. The cost per identification of a true relapse was approximately US $6000. (**B**) Costs of the components of follow-up. This figure illustrates the costs per patient of the follow-up visits, the CXRs and the CTs for each of the first 6 years of follow-up. A total of 64% of the total CT costs were incurred during the first year of follow-up, as opposed to 40% of the cost of the follow-up visits. (**C**) Routine costs *vs* investigations of suspected relapse. This figure illustrates the routine costs of follow-up and the costs of the investigations of suspected relapse for each of the first 6 years of follow-up. Routine follow-up costs amounted to 85% of the total cost of follow-up.

In all, 51% of the total cost of follow-up was attributable to the visits themselves, 39% to the CT scans and 10% to the chest X-rays (Figure 3A

). The cost per identification of a true relapse was US $6000. The cost of follow-up was highest during the first year during which CT scans were responsible for half of the follow-up expenses (Figure 3B). A total of 84% of the costs of follow-up was incurred by routine tests, while 15% was due to the investigation of suspected relapses (Figure 3C). Routine CT scans alone accounted for 29% of the total cost of follow-up.

## DISCUSSION

Our review of follow-up of patients with Hodgkin's disease after potentially curative therapy found that (1) two-thirds of relapses were identified clinically; (2) routine radiological tests identified a quarter of all relapses, yet accounted for half of the total costs of follow-up; (3) 211 routine CT scans were performed which detected only two of 22 relapses, yet accounted for 29% of the total follow-up costs; (4) for every true relapse identified, four false relapses were investigated, yet the majority of the cost of follow-up (84%) was incurred by the cost of routine follow-up; and that (5) prior treatment with radiotherapy alone made a suspected relapse more likely to be a true relapse; other factors, including the modality of the suspected relapse, were unhelpful.

The attribution of suspected relapses to either the patient, the physician, a radiological investigation or a laboratory value may not have adequately categorised cases in which a subtle radiological abnormality, in itself insufficient to drive further investigations, nevertheless bolstered a physician's decision to investigate a clinical symptom. In such a case, the suspected relapse would be attributed to the patient alone without reflecting the role played by the radiological abnormality. Our categorisation of relapses relied mainly on a careful reading of the physicians' follow-up visit notes and in the vast majority of cases, a single modality appeared to trigger a suspected relapse in a decisive manner. It may also be argued that the hierarchical grading system that we used biased against radiological investigations that identified pathology concurrently with the appearance of clinical symptoms or signs. However, in only one of 22 true relapses did such a situation occur (the patient reported a retrosternal ache and the chest X-ray demonstrated a 4 cm anterior mediastinal mass). In addition, only 8% of our patients had Ann Arbor stage IV disease and only 10% had an IPS of ⩾3. Our results may therefore be less readily generalised to patients with high-risk disease.

The relapse rate in this series (21%) is comparable to that found by Radford *et al* (18%; Radford *et al*, 1997) and Torrey *et al* (22%; Torrey *et al*, 1997) in their reviews of Hodgkin's follow-up. A total of 64% of relapses in our series were identified clinically, compared to 69% in the series of Torrey *et al*. In the series of Radford *et al*, 81% of relapses were identified through symptoms alone, but the extent of radiological investigations was limited to a chest X-ray if the patient had mediastinal disease at presentation. The extent to which radiographs contributed to the cost of follow-up in our series (49%) is similar to that reported by Torrey *et al* (62%). No other series has reported the rates and modalities of false relapses nor the extent to which CT scans help detect relapses.

For the exclusive purpose of detecting relapse, it would make sense to tailor the frequency of follow-up to the likelihood of relapse based on the treatment modality and the IPS. However, the follow-up process serves many purposes, including the detection and management of the late toxicities of therapy and the provision of psychosocial support. We favour a common follow-up frequency that provides equal psychosocial support to all Hodgkin's patients and is easier to implement logistically. While some have advocated fewer follow-up visits and more patient education on the grounds that the identification of most relapses stem from patient-reported symptoms (Radford *et al*, 1997), more than half of the relapses in our series were detected through the physician's clinical assessments, radiological investigations or complete blood counts, all of which require routine visits. Our experience supports a follow-up frequency based on the Cotswolds recommendations (Lister *et al*, 1989).

On the other hand, our series demonstrates that the routine use of the CT scan is both costly and of little value in the identification of relapse. The routine CT scan's ability to generate six times more false relapses stems from the diagnostic difficulty posed by residual masses after completion of therapy. Our results argue that the elimination of routine follow-up CT scans would translate into a significant reduction in resource utilisation and unnecessary radiation exposure without compromising the follow-up of patients with Hodgkin's disease.
